# Intraoperative challenges and complications of cataract surgery between cataract surgery alone and phacovitrectomy in eyes with diabetic retinopathy: efficacy of illuminated chopper-assisted cataract surgery

**DOI:** 10.1186/s12886-023-02982-6

**Published:** 2023-05-26

**Authors:** Sung Ha Hwang, Haram Kim, Dae Yeong Lee, Dong Heun Nam

**Affiliations:** Department of Ophthalmology, Gil Medical Center, College of Medicine, Gachon University, 21 Namdong-daero 774-beon-gil, Namdong-gu, Incheon, 21565 Republic of Korea

**Keywords:** Diabetic retinopathy, Phacovitrectomy, Intraoperative challenges, Illuminated chopper-assisted cataract surgery

## Abstract

**Background:**

To compare the intraoperative challenges, complications, and operation time of illuminated chopper-assisted cataract surgery between cataract surgery only and phacovitrectomy in eyes with diabetic retinopathy.

**Methods:**

One university hospital, retrospective case series. Two hundred ninety-five eyes of 295 consecutive patients with diabetic retinopathy who underwent cataract surgery only or phacovitrectomy were retrospectively reviewed. Intraoperative challenges and complications of cataract surgery were thoroughly analyzed by 3D viewing of digitally recorded videos. The pupil diameter, operation time, and improved efficacy (100/operation time × pupil diameter) were compared between the cataract surgery only and phacovitrectomy groups.

**Results:**

Of the 295 eyes, 211 underwent cataract surgery only, and 84 underwent phacovitrectomy. Intraoperative challenges such as small pupil, miosis, or poor red reflex occurred more frequently (46 [21.8%] vs. 28 [33.3%], p = 0.029); pupil diameter was smaller (7.34 ± 0.94 vs. 6.89 ± 0.88 mm, p < 0.001) in the phacovitrectomy group than in the cataract surgery only group; however, rates of posterior capsule rupture and operation time were not different between the two groups (0 [0%] vs. 1 [1.2%], p = 0.285; 16.54 ± 2.65 vs. 16.31 ± 4.30 min, p = 0.434). Improved efficacy was higher in the phacovitrectomy group (0.85 ± 0.18 vs. 0.97 ± 0.28, p = 0.002).

**Conclusions:**

The use of an illuminated chopper is a potential solution for diabetic cataract surgery, particularly in phacovitrectomy, by decreasing the use of supplemental devices, operation time, and posterior capsule rupture.

**Trial registration:**

Retrospectively registered.

**Supplementary Information:**

The online version contains supplementary material available at 10.1186/s12886-023-02982-6.

## Background

Given the demographic trends in diabetes and cataracts, cataract surgeons are likely to encounter patients with both comorbidities at an increasing frequency. Cataract surgery in patients with diabetes may be associated with intraoperative surgical challenges (small pupils or advanced cataracts) and an increased risk of intraoperative complications (posterior capsule rupture). However, there are few studies that specifically analyzed the intraoperative complications of diabetic cataract surgery. Recent large sample-based studies have demonstrated that intraoperative difficulties and posterior capsule ruptures were almost twice as common in patients with diabetic retinopathy (DR) [1–3].

With the advances in instruments and techniques, combined cataract surgery and vitrectomy (phacovitrectomy) is gaining popularity. Phacovitrectomy offers several advantages such as fast visual recovery, avoidance of cataract surgery in the vitrectomized eye, and low cost [4–7]. Nevertheless, it presents challenges such as long operation time and high rate of intraoperative anterior chamber instability, miosis, or posterior capsule rupture [8, 9]. Therefore, cataract surgery in phacovitrectomy for DR may be more challenging than diabetic cataract surgery alone. Although phacovitrectomy is increasingly accepted in eyes with DR, there have been few studies on the intraoperative challenges and complications of cataract surgery in phacovitrectomy for DR [10, 11].

Intracameral illuminator-assisted cataract surgery has been combined with vitrectomy in eyes with poor red reflex [12, 13]. An illuminated chopper (Nam illumination probe with chopper, Oculight) was approved by the US FDA for improved visualization and manipulation during cataract surgery [14, 15]. In this study, we compared the challenges, complications, and operation time during cataract surgery between cataract surgery alone and phacovitrectomy in eyes with DR to evaluate the efficacy of illuminated chopper-assisted cataract surgery.

## Methods

Electronic medical records of consecutive patients who were diagnosed with DR and underwent cataract surgery only or phacovitrectomy at one university hospital between July 1, 2020, and May 31, 2022, were reviewed. This study was approved by the Institutional Review Board of our university (IRB number: GCIRB2022-261). The study conformed to the tenets of the Declaration of Helsinki. Based on the disclosure of conflicts of interest related to this study, the Conflict of Interest Review Board of our university thoroughly investigated the data collection and patient selection.

**Fig. 1:**
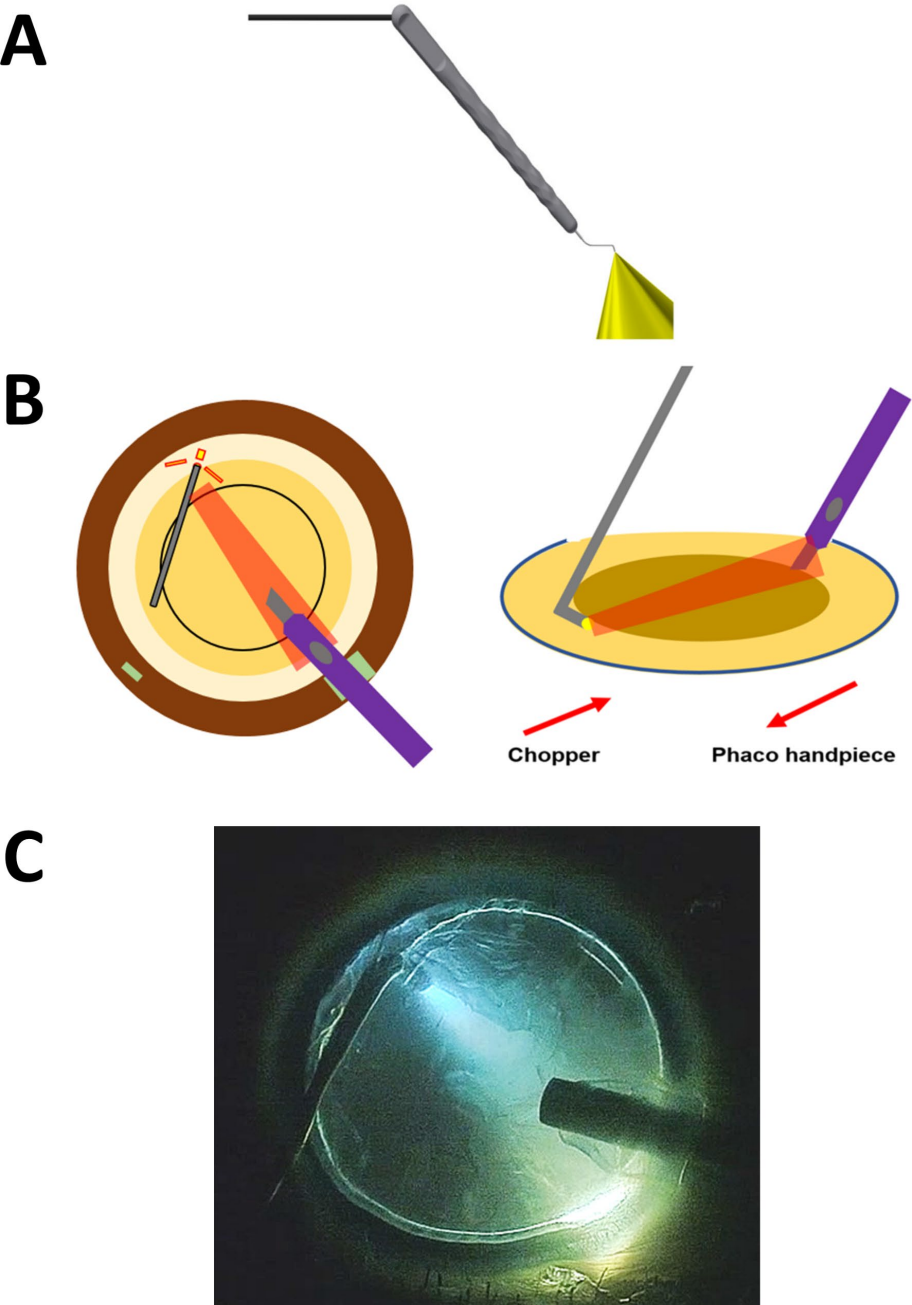
**Illustration of illuminated-chopper**. (**A**) iChopper, Nam illumination probe with a chopper, Korea, and USA Food and Drug Administration cleared (Oculight, South Korea). (**B**) Phaco chop using an illuminated chopper. (**C**) Still video image of illuminated chopper during phaco chop

Both cataract surgery only (Signature PRO, Johnson & Johnson) under sub-tenon anesthesia and phacovitrectomy (Constellation, Alcon) under retrobulbar anesthesia were performed by an experienced surgeon (D.H.N.) using a 3D heads-up visualization system (Ngenuity, Alcon). We used the same standardized settings on both two different machines: 33 cc/min maximum aspiration rate, 340mmHg maximum vacuum and 50% maximum phacoemulsification power. The novel cataract surgery technique using an illuminated chopper (Nam illumination probe with chopper, Oculight) described in our previous studies was used in the consecutive, interventional case series (Fig. 1, see Additional file 1, which demonstrated the phaco-chop with illuminated chopper) [16–20]. Cataract surgery in phacovitrectomy was performed after 25 gauge 3-port trocar cannula insertion. In cases of increased posterior pressure due to retrobulbar anesthesia, minimal core vitrectomy was performed before cataract surgery. Four flexible polymer iris hooks (Alcon/Grieshaber) and trypan blue staining were performed when required.

The preoperative data used for analysis were age, sex, laterality, axial length, anterior chamber depth, intraocular pressure (IOP), corrected distance visual acuity, and endothelial cell density. Visual acuity was measured logarithmically using the minimum angle of resolution (logMAR) notation. Additionally, information about pseudoexfoliation, tamsulosin use, history of intravitreal injection, laser treatment, or uveitis was collected to assess the risk of complications in cataract surgery.

According to the ETDRS criteria, DR was divided into four stages: mild, moderate, severe non-proliferative DR, and proliferative DR. Nuclear sclerosis values for each case were derived by calculating the average nuclear opalescence and nuclear color grading, as described in the Lens Opacities Classification System III [21]. Presences of cortical opacity and anterior and posterior subcapsular opacity were also counted.

Intraoperative challenges and complications of cataract surgery were thoroughly reviewed through 3D viewing of digitally recorded surgical videos (Ngenuity, Alcon). The intraoperative anterior segment data elements used for analysis were operation time, pupil size, iris events, other intraoperative events, and the use of supplemental instruments, such as iris hooks or trypan blue dye. The operation time of cataract surgery was measured from incision to wound closure. The pupil size was calculated by measuring the mean pupil diameter during phacoemulsification. We used the ImageJ software program (version 1.51j8, NIH, USA) to measure the pupil diameter in pixels from video still images. The pixel measurements were then converted into millimeters using the constant limbus size as a reference. Iris events included small pupils (< 6 mm), intraoperative miosis (invisible capsulorhexis margin), floppy iris, incarceration or prolapse of the iris, and posterior synechiae. Other intraoperative events include poor red reflex, tears of the posterior and anterior capsule, zonular weakness, and dropped lens material.

The postoperative data elements used for analysis were visual acuity, IOP, and endothelial cell count at one month postoperatively. Posterior segment complications, such as retinal tears or cystoid macular edema, were not considered in this study.

The main outcome measures were intraoperative challenges and complications of the anterior segment, operative time, pupil size, and improved efficacy. Cataract surgery takes longer for eyes with small pupils than for eyes with large pupils [22, 23]. If the visibility of the surgical field is increased by using the illuminated chopper, the operating time will not be increased. Thus, the improved efficacy score in eyes with small pupils by the illuminated chopper was defined as the inverse of the mean pupil size (100/surgical time [min] × pupil size [mm]).

The Mann–Whitney U test, Pearson’s chi-square, and Fisher’s exact tests were used to test the significance of the differences in variables between the cataract surgery only and phacovitrectomy groups. Statistical significance was set at p < 0.05. All statistical analyses were performed using the SPSS software (version 22.0, IBM Corp).

## Results

Two hundred ninety-five eyes of 295 patients with DR were included in this study. Of the 295 eyes, 211 underwent cataract surgery only, and 84 underwent phacovitrectomy. Patients were younger (66.32 vs. 58.20, p < 0.001), and baseline visual acuity (logMAR) was worse (0.41 vs. 0.97, p < 0.001) in the phacovitrectomy group than in the cataract surgery only group. Furthermore, the stage of DR was more severe (PDR: 37.6% vs. 75.0%), and a previous history of intravitreal injection (19.4% vs. 66.7%, p < 0.001) and laser treatment (6.2% vs. 19.0%, p = 0.002) were more common in the phacovitrectomy group. However, there were no significant differences in axial length, cataract grade, tamsulosin use, pseudoexfoliation, or endothelial cell count (Table 1).

**Table 1:** Baseline demographics

	Cataract surgery	Phacovitrectomy	P Value
Number of eyes (n)	211	84	
Mean age (year) ± SD	66.32 ± 10.08	58.20 ± 12.40	< 0.001
Sex, (M:F)	107:104	55:29	0.027
Laterality (right:left)	103:108	42:42	0.898
Mean AL (mm) ± SD	23.56 ± 1.01	23.91 ± 1.71	0.082
Mean ACD (mm) ± SD	3.10 ± 0.43	3.14 ± 0.38	0.448
Characteristics of cataract			
NS grade, Mean ± SD	3.55 ± 1.20	3.31 ± 0.91	0.100
Presence of CO, n (%)	135 (64.0%)	58 (69.0%)	0.498
Presence of PSCO, n (%)	41 (19.4%)	13 (15.5%)	0.506
Mature cataract, n (%)	8 (3.8%)	3(3.6%)	0.615
Diabetic retinopathy stage, n (%)			< 0.001
Mild NPDR	112 (53.1%)	13 (15.5%)	
Moderate NPDR	36 (17.1%)	6 (7.1%)	
Severe NPDR	25 (11.8%)	2 (2.4%)	
PDR	38 (37.6%)	63 (75.0%)	
Presence of ME	26 (12.3%)	27 (32.1%)	< 0.001
Tamsulosin use, n (%)	13 (6.2%)	1 (1.2%)	0.124
Pseudoexfoliation, n (%)	3 (1.4%)	1 (1.2%)	0.679
Previous history			
Uveitis, n (%)	3 (1.4%)	1 (1.2%)	0.679
Intravitreal injection	41 (19.4%)	56 (66.7%)	< 0.001
Mean number of IVIs	0.36 ± 0.94	1.16 ± 1.35	< 0.001
Laser treatment	13 (6.2%)	16 (19.0%)	0.002

ACD, anterior chamber depth; AL, axial length; CO, cortical opacity; IVI, intravitreal injection; ME, macular edema; NPDR; nonproliferative diabetic retinopathy; NS, nuclear sclerosis; PDR, proliferative diabetic retinopathy; PSCO, posterior subcapsular cortical opacity

Among the 84 eyes that underwent phacovitrectomy, vitreous hemorrhage was the most common cause of vitrectomy (48 eyes, 57.1%). Diabetic macular edema (14 eyes, 16.7%), tractional retinal detachment (18 eyes, 21.4%), and epiretinal membranes (21 eyes, 25.0%) were observed. In addition, there were other causes, such as vitreous opacity (5 eyes, 6.0%), rhegmatogenous retinal detachment (1 eye, 1.2%), macular hole (1 eye, 1.2%), and myopic macular degeneration (2 eyes, 2.4%) (Table 2).

**Table 2:** Vitreoretinal indications for phacovitrectomy

Vitreoretinal disease	Number of eyes (n)	Incidence (%)
Vitreous hemorrhage	48	57.1
Diabetic macular edema	14	16.7
Tractional retinal detachment	18	21.4
Epiretinal membrane	21	25.0
Vitreous opacity	5	6.0
Rhegmatogenous retinal detachment	1	1.2
Macular hole	1	1.2
Myopic macular degeneration	2	2.4

Small pupils (< 6 mm) (16 [7.6%] vs. 13 [15.5%], p = 0.036), intraoperative miosis (7 [3.3%] vs. 8 [9.5%], p = 0.033), and a poor red reflex (21 [10.0%] vs. 12 [14.3%], p = 0.193) were more prevalent in the phacovitrectomy group. Overall, more eyes with at least one intraoperative event were found in the phacovitrectomy group (46 (21.8%) vs. 28 (33.3%), p = 0.029). Nonetheless, the rates of intraoperative complications were very low, and there was no significant difference in complications between the two groups (3 [1.4%] vs. 3 [3.6%], p = 0.226). In the cataract surgery only group, an anterior capsule tear and zonular dialysis occurred in two (0.9%) and one eye (0.5%), respectively. In the phacovitrectomy group, posterior capsule rupture, zonular dialysis, and dropped lens material occurred in one eye (1.2%), two eyes (2.4%), and one eye (1.2%), respectively (Table 3).

**Table 3:** Intraoperative challenges and complications of anterior segment

	Cataract surgery (n = 211)	Phacovitrectomy (n = 84)	P Value
Intraoperative challenges, n (%)			
Small pupil (< 6 mm)	16 (7.6%)	13 (15.5%)	0.036
Constriction (miosis)	7 (3.3%)	8 (9.5%)	0.033
Floppy iris	9 (4.3%)	1 (1.2%)	0.170
Incarceration or prolapse	5 (2.4%)	3 (3.6%)	0.409
Posterior synechiae	0 (0%)	2 (2.4%)	0.080
Poor red reflex	21 (10.0%)	12 (14.3%)	0.193
Number of eyes with intraoperative challenges	46 (21.8%)	28 (33.3%)	0.029
Complications, n (%)			
Posterior capsule tear	0 (0%)	1 (1.2%)	0.285
Anterior capsule radial tear	2 (0.9%)	0 (0%)	0.511
Zonular dialysis	1 (0.5%)	2 (2.4%)	0.196
Dropped lens material	0 (0%)	1 (1.2%)	0.285
Number of eyes with complications	3 (1.4%)	3 (3.6%)	0.226

The mean pupil diameter during phacoemulsification was significantly smaller in the phacovitrectomy group (7.34 ± 0.94 mm vs. 6.89 ± 0.88 mm, p < 0.001), but there was no difference in mean operation time between the two groups (16.54 ± 2.65 min vs. 16.31 ± 4.30 min, p = 0.434). Iris hooks and capsule staining were not used in either group. When the improved efficacy was calculated by applying the formula mentioned in the method, the value was significantly higher in the phacovitrectomy group (0.85 ± 0.18 vs. 0.97 ± 0.28, p = 0.002) (Table 4).

**Table 4:** Intraoperative parameters of anterior segment

	Cataract surgery (n = 211)	Phacovitrectomy (n = 84)	P Value
Mean operation time (minute) ± SD	16.54 ± 2.65	16.31 ± 4.30	0.434
Mean pupil diameter (mm) ± SD	7.34 ± 0.94	6.89 ± 0.88	< 0.001
Mean improved efficacy			
[100/pupil diameter(mm)*operation time(minute)]	0.85 ± 0.18	0.97 ± 0.28	0.002
Supplemental tools			
Iris hooks	0 (0%)	0 (0%)	N/A
Anterior capsule staining	0 (0%)	0 (0%)	N/A

N/A, not applicable

Because the indications for surgery performed in the two groups were different, both baseline and postoperative visual acuities (logMAR) were worse (0.41 vs. 0.97, p < 0.001; 0.21 vs. 0.52, p < 0.001) in the phacovitrectomy group. Nevertheless, there was no difference in IOP or endothelial cell count between the two groups before and after surgery (Table 5).

**Table 5:** Clinical outcomes

		Cataract surgery (n = 211)	Phacovitrectomy (n = 84)	P value
CDVA (logMAR) ± SD	Pre	0.41 ± 0.52	0.97 ± 0.87	< 0.001
	Post	0.21 ± 0.42	0.52 ± 0.80	< 0.001
IOP (mmHg)	Pre	13.15 ± 3.50	13.26 ± 4.00	0.811
	Post	12.85 ± 3.45	13.42 ± 4.05	0.075
Endothelial cell density (/$${mm}^{2})$$± SD	Pre	2364.36 ± 345.21	2451.34 ± 363.92	0.062
	Post	2243.14 ± 514.54	2343.27 ± 384.28	0.119

CDVA, corrected distant visual acuity; IOP, intraocular pressure

## Discussion

Although phacovitrectomy is increasingly accepted in eyes with DR, there are more concerns about the intraoperative challenges and complications of cataract surgery in phacovitrectomy for DR [6, 7, 13, 24, 25]. In terms of the method of anesthesia, insertion of a sclerotomy cannula, stage of DR, or previous intravitreal injections, there are differences between cataract surgery only and cataract surgery in phacovitrectomy. Nonetheless, it is unclear whether there is any difference in the rates of intraoperative challenges, complications, and operation time of cataract surgery between cataract surgery only and phacovitrectomy in eyes with DR.

In the present study, we compared the operation time, intraoperative challenges, and complications of diabetic cataract surgery between cataract surgery only and phacovitrectomy, in which a single surgeon performed all vitrectomies and cataract surgeries using a 3D heads-up visualization system. In particular, we thoroughly observed intraoperative challenges and complications of cataract surgery in both groups through 3D viewing of digitally recorded surgical videos.

In eyes with DR, the rates of posterior capsule rupture were reportedly 1.4–2.3%, twice as common as in non-DR eyes. Even for senior surgeons, the rate of posterior capsule rupture was 1.6% [1–3]. In the cataract surgery only group of this study, the rate of intraoperative challenges including small pupil, miosis, or poor red reflex was 21.8%, which was higher than the rate (11.1%) of intraoperative difficulties such as mechanical pupil dilation or staining of the anterior capsule in large sample-based studies. However, we did not encounter any posterior capsule ruptures in the cataract surgery only group, even without any mechanical pupil dilation or staining of the lens capsule. The use of an illuminated chopper may have improved efficacy in diabetic cataract surgeries and then simplified the challenging surgeries [14, 16, 17].

In the phacovitrectomy group in this study, the rate of intraoperative challenges, including small pupil, miosis, or poor red reflex, was 33.3%, which was significantly higher than that in the cataract surgery only group (21.8%, p = 0.029). Furthermore, the pupil diameter was smaller in the phacovitrectomy group than in the cataract only group (7.34 ± 0.94 mm vs. 6.89 ± 0.88 mm, p < 0.001). Therefore, cataract surgery in phacovitrectomy could have taken a longer operation time and had more complications, such as posterior capsule rupture. However, there was no difference in operation time between the two groups (16.54 ± 2.65 min vs. 16.31 ± 4.30 min, p = 0.434). The rate of posterior capsule rupture was very low (1.2%) in the phacovitrectomy group, even without mechanical pupil dilation or staining of the lens capsule. These favorable outcomes may be related to higher improved efficacy in the phacovitrectomy group (0.85 ± 0.18 vs. 0.97 ± 0.28, p = 0.002) [12, 13].

**Fig. 2:**
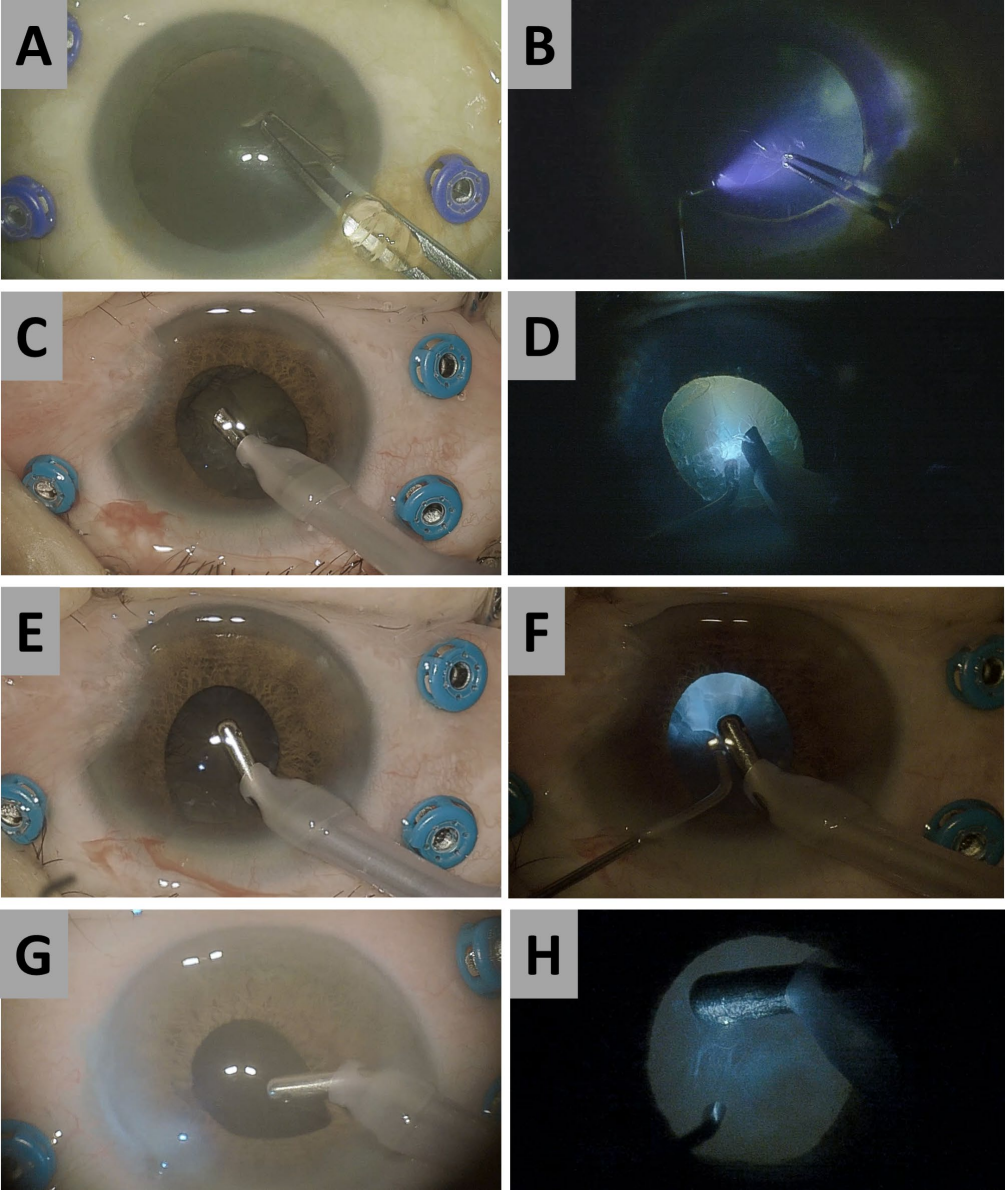
**Photographs comparing the conventional microscopic and illuminated chopper-assisted views during cataract surgery in phacovitrectomy**. (**A**, **B**) A 61-year-old female with poor red reflex due to severe vitreous hemorrhage. The capsulorhexis flap and margin were better visible when using illuminated chopper. (**C**, **D**, **E**, **F**) A 57-year-old male with small pupil and floppy iris. Phacoemulsification and irrigation and aspiration progressed easily with improved efficacy of lens by illuminated chopper. (**G**, **H**) An 83-year-old female with intraoperative miosis and iris prolapse. Polishing capsule was done safely with illuminated chopper

Regarding intraoperative complications, we found a very low risk of posterior capsule rupture in both the cataract surgery only and phacovitrectomy groups. Intraoperative challenges such as small pupil size, miosis, or poor red reflex were resolved using an illuminated chopper in challenging cataract surgeries, even without any mechanical pupil dilation or staining of the lens capsule (Fig. 2). Therefore, the operation times were not prolonged in either group.

This study was limited by the experienced single-surgeon, retrospective design, which may have resulted in bias in the outcome analyses. Additional research may be necessary to determine whether general ophthalmologists could demonstrate the benefits of the illuminated chopper found in this study. Although a randomized controlled study is the gold standard level of evidence, it is difficult to conduct such a study on diabetic cataract surgery. A pilot study performed by the Diabetic Retinopathy Clinical Research Network was terminated early due to difficulty recruiting enough participants for such a prospective study [26]. However, this is the first study to compare intraoperative challenges and complications during cataract surgery between cataract surgery only and phacovitrectomy in eyes with DR. Furthermore, a unique advantage of this study is the accurate and complete capture of intraoperative challenges such as small pupil, miosis, or poor red reflex through 3D review of digitally recorded surgical video. Our data will allow for more accurate surgical planning and counseling of patients regarding the intraoperative difficulties of diabetic cataract surgery, especially in diabetic phacovitrectomy.

## Conclusions

In conclusion, intraoperative challenges of diabetic cataract surgery, such as small pupil, miosis, or poor red reflex, were substantial in both the cataract surgery only and phacovitrectomy groups, while it was more common in the phacovitrectomy group. Even without any mechanical pupil dilation or staining of the lens capsule, the rates of posterior capsule rupture were very low and operation times were not long in either group. Patients with cataract and DR should be offered preoperative counseling on the risk of intraoperative challenges, and surgeons should use the illuminated chopper during the challenging cataract surgery to avoid the use of supplemental devices such as mechanical pupil dilation or staining of the lens capsule and prevent intraoperative complications.

## Electronic supplementary material

Below is the link to the electronic supplementary material.

Supplementary Material 1

## Data Availability

The datasets used and/or analyzed during the current study are available from the corresponding author on reasonable request.

## List of Abbreviations

DR: Diabetic retinopathy
IOP: Intraocular pressure
logMAR: Logarithmically using the minimum angle of resolution
